# Interaction of 6 Mercaptopurine with Calf Thymus DNA – Deciphering the Binding Mode and Photoinduced DNA Damage

**DOI:** 10.1371/journal.pone.0093913

**Published:** 2014-04-09

**Authors:** Sayeed Ur Rehman, Zahid Yaseen, Mohammed Amir Husain, Tarique Sarwar, Hassan Mubarak Ishqi, Mohammad Tabish

**Affiliations:** 1 Department of Biochemistry, Faculty of Life Sciences, A.M. University, Aligarh, Uttar Pradesh, India; 2 Department of Chemistry, Faculty of Sciences, A.M. University, Aligarh, Uttar Pradesh, India; University of Quebect at Trois-Rivieres, Canada

## Abstract

DNA is one of the major intracellular targets for a wide range of anticancer and antibiotic drugs. Elucidating the binding between small molecules and DNA provides great help in understanding drug-DNA interactions and in designing of new and promising drugs for clinical use. The ability of small molecules to bind and interfere with DNA replication and transcription provides further insight into how the drugs control the expression of genes. Interaction of an antimetabolite anticancer drug 6mercaptopurine (6MP) with calf thymus DNA was studied using various approaches like UV-visible spectroscopy, fluorescence spectroscopy, CD, viscosity and molecular docking. UV-visible spectroscopy confirmed 6MP-DNA interaction. Steady state fluorescence experiments revealed a moderate binding constant of 7.48×10^3^ M^−1^ which was consistent with an external binding mode. Competitive displacement assays further confirmed a non-intercalative binding mode of 6MP which was further confirmed by CD and viscosity experiments. Molecular docking further revealed the minimum energy conformation (−119.67 kJ/mole) of the complex formed between DNA and 6MP. Hence, the biophysical techniques and *in-silico* molecular docking approaches confirmed the groove binding/electrostatic mode of interaction between 6MP and DNA. Further, photo induced generation of ROS by 6MP was studied spectrophotometrically and DNA damage was assessed by plasmid nicking and comet assay. There was a significant increase in ROS generation and consequent DNA damage in the presence of light.

## Introduction

Interaction between drug molecules and DNA has become an active area of research in recent years [1]–[2]. DNA is one of the most important bio-macromolecule since it controls the structure and function of the cell. It is also a major intracellular target for a wide range of anticancer and antibiotic drugs [3]–[4]. Several studies have been conducted to elucidate the binding of these small molecules and DNA that provide great help in understanding drug-DNA interaction and in designing of new and promising drugs for clinical use. The ability of small molecules to bind and interfere with DNA replication and RNA transcription provides further insight into how the drugs control the expression of genes [5]–[7]. Interaction of small molecules and DNA are mainly of two types, covalent interactions and non-covalent interactions. Three major modes of non-covalent interactions are electrostatic interactions, groove binding and intercalative binding. Small molecule can interact with DNA involving a single mode of binding or mixed binding modes. It is worth noting that the property of mixed binding mode can be linked to their mechanism of action and therapeutic efficiency [8], . Studying DNA as a drug target is attractive due to the availability of the well-studied three-dimensional DNA structures and the predictability of their accessible chemical functional groups. However, the number of known DNA-based drug targets is still very limited in comparison to the protein-based drug targets [10].

6 Mercaptopurine (6MP) is an anticancer drug commonly used to treat childhood acute lymphoblastic leukemia. It is also used as an immunosuppressive as well as anti inflammatory drug. 6MP comes under the antimetabolite class of anticancer drug that leads to inhibition of purine *de novo* synthesis. 6MP metabolism in cell leads to the formation of 6 thioguanine (6-TG) that further gets incorporated in DNA. After being methylated, 6meTG mispairs with thymine (T) in subsequent replications that invokes mismatch repair system which is lethal [11]. 6MP is also found to control the expression of various genes in cell where it up-regulate certain genes (*abcc4, xdh, krt2-7*) or down-regulate some genes (*abcc8, Hoxa13*) [12], [13]. Although their metabolism has been studied in detail, the precise molecular events that underlie their therapeutic activity have remained unclear. Due to direct or indirect interaction of 6MP with different genes/products, study of 6MP-DNA interaction under physiological conditions was of high significance.

In the present study we have evaluated the interaction of 6MP and DNA in vitro by using various biophysical techniques and *in-silico* by exploiting molecular docking. UV-visible spectroscopy was used to determine the binding mode as well as the stability of DNA-drug complex [14]–[16]. Steady state fluorescence, circular dichroism (CD) spectroscopy and viscosity experiments were employed to get into the insight of drug-DNA interaction. Molecular docking further revealed the minimum energy isomer of the complex formed between DNA and 6MP. Several studies were also conducted to assess the DNA damage caused by 6MP induced ROS generation in presence of light.

## Materials and Methods

### Materials

6 Mercaptopurine (6MP), calf thymus DNA (CT-DNA), acridine orange (AO) and Hoechst 33258 were purchased from Sigma Aldrich, USA. Ethidium bromide (EB) was purchased from Himedia, India. Plasmid pBR322 was purified according to method described earlier [17]. All the other chemicals and solvents were of reagent grade and used without purification.

### Sample Preparation

Stock solution of 6MP was prepared in DMSO. CT-DNA was suspended in 10 mM Tris-HCl buffer (pH 7.2) at 4°C for 24 h with occasional mixing by vortex to ensure the formation of a homogeneous solution. To check the purity of DNA solution, absorbance ratio A_260_/A_280_ was recorded. No further purification was required since the attenuance ratio was between 1.8 and 1.9. Various concentration of DNA solutions were used in different experiments after determining its concentration spectrophotometrically using average molar extinction coefficient value of 6600 M^−1^ cm^−1^ of a single nucleotide at 260 nm. All reactions were done in presence of 10 mM Tris-HCl buffer (pH 7.2) at room temperature.

### UV-visible Spectroscopy

UV-visible spectra were recorded on Beckman DU 40 spectrophotometer (USA) using a cuvette of 1×1cm path length. Spectra of 6MP and 6MP-DNA complex were measured in the wavelength range of 300–400 nm. Increasing concentration of CT-DNA was titrated against 25 μM of 6MP.

### Fluorescence Studies

Fluorescence emission spectra of 6MP were recorded on a Shimadzu spectroflurometer-5000 (Japan) equipped with xenon flash lamp using 1.0 cm quartz cells. Excitation was fixed at 280 nm [18] and emission spectra were recorded from 300 nm to 500 nm after setting the widths of both the excitation and the emission slits at 10 nm. Appropriate blanks corresponding to the buffer were subtracted to correct the background fluorescence. The fluorescence titration was carried out by keeping the concentration of 6MP constant (50 μM) and varying DNA concentration (0–45 μM). In case of EB displacement assay, a solution containing 2 μM of EB and 20 μM of DNA was titrated with increasing concentration of 6MP. EB-DNA complex was excited at 471 nm and emission spectra were recorded from 500–700 nm. In another experiments, AO-DNA complex was excited at 490 nm while DNA-Hoechst 33258 complex was excited at 343 nm and emission spectra were recorded from 500–600 nm and 360–600 nm respectively. Iodide quenching experiments were performed in presence and absence of DNA. Emission spectra were recorded either in presence or absence of 50 μM DNA in 3ml reaction mixture which included 50 μM 6MP, 10 mM Tris-HCl (pH 7.2) and varying concentration of KI between 0–8 mM. Excitation was done at 280 nm and emission spectra were recorded from 300–500 nm. Effect of ionic strength was studied by varying the concentration of NaCl between 0–70 mM in total volume of 3 ml containing 50 μM 6MP, 50 μM CT-DNA and 10 mM Tris-HCl (pH 7.2). Excitation was done at 280 nm and emission spectra were recorded between 300–500 nm.

### Circular Dichroism (CD) Studies

CD spectra of DNA alone and 6MP-DNA complex were recorded using Applied Photophysics CD spectrophotometer (model CIRASCAN, U.K.) equipped with a Peltier temperature controller to keep the temperature of the sample constant at 25°C. All the CD spectra were recorded in a range from 200 nm to 320 nm with a scan speed of 200 nm/min with a spectral band width of 10 nm. Average of three scans was taken in all experiments. Background spectrum of buffer solution (10 mM Tris-HCl, pH 7.2) was subtracted from the spectra of DNA and 6MP-DNA complex.

### Viscosity Measurement

To further elucidate the binding mode of 6MP, viscosity measurements were carried out by keeping DNA concentration constant (100 μM) and varying the concentration of 6MP. Viscosity measurements were carried out with an Ubbelohde viscometer (Cannon, Model-9721-K56, Cole-Parmer, USA) suspended vertically in a thermostat at 25°C (accuracy +0.1°C). The flow time was measured with a digital stopwatch, and each sample was tested three times to get an average calculated time. The data were presented as (η/η_0_) versus ratio of DNA/6MP concentrations, where η and η_0_ are the viscosity of DNA in the presence and absence of 6MP.

### Molecular Docking

HEX 6.3, a molecular graphics program, was used to study the 6MP-DNA interaction. Structure of the B–DNA dodecamer d(CGCGAATTCGCG)_2_ (PDB ID: 1BNA) was downloaded from the protein data bank (http://www.rcsb.org./pdb). Mol file of 6MP was obtained from http://www.drugbank.ca/drugs/DB01033 and further converted into PDB format using Avogadro’s 1.01. The Hex 6.3 performs docking using Spherical Polar Fourier Correlations. It necessitates the ligand and the receptor as input in PDB format. The parameters that were used for docking include: correlation type – shape only, FFT mode –3D, grid dimension –0.6, receptor range –180, ligand range –180, twist range –360, distance range –40. PyMol software was used for visualization of the docked pose.

### ROS Generation and DNA Damage Assessment

#### Superoxide generation assay

Superoxide generation by 6MP was studied by nitroblue tetrazolium (NBT) reduction assay [19]. Assay mixture contained 10 mM sodium phosphate buffer (pH 7.8), 0.5 mM NBT, 0.1 mM EDTA and 0.06% Triton X-100 in a total reaction mixture of 3 ml. After mixing, samples were placed in front of white fluorescent lamp at a distance of 10 cm and absorbance was recorded at 560 nm using a suitable blank. Concentration dependent generation of superoxide in presence of white light was studied. There was no observable change in the temperature of the solution at the end of experiment.

### Plasmid Nicking Assay

To examine the generation of nicks in double stranded DNA by ROS generated by 6MP, plasmid nicking assay was performed. Reaction mixture contained 0.5 μg pBR322 plasmid DNA, desired concentration of 6MP and 10 mM Tris-HCl (pH 7.2) was added to a final volume of 25 μl in all the tubes. Irradiation was performed with white light for 4 h at 37°C. After incubation, 5 μl of 6X tracking dye solution containing (40 mM EDTA, 0.05% bromophenol blue and 50% glycerol) was added and the reaction mixture was subjected to electrophoresis using 1% (w/v) agarose gel. Gel was stained with EB and photographed on a UV-transilluminator.

### Comet Assay

DNA damage caused by 6MP in dark, and in presence of light was analysed using comet assay. Fresh blood samples (3ml) were obtained from healthy volunteers by vein puncture and stored in presence of heparin to avoid clotting. Lymphocytes were isolated from the diluted blood using Histopaque 1077 (HiMedia) and suspended in RPMI 1640. Trypan Blue Exclusion test [20] was performed before the start and at the end of experiment to check the viability of lymphocytes. Lymphocytes (1×10^5^ cells) were exposed to different concentrations of 6MP in dark and in presence of light in a total reaction volume of 500 μl that also included Ca^2+^ and Mg^2+^ free PBS and RPMI 1640. Incubation was performed for 2 h at 37°C and the mixture was centrifuged at 5000 rpm to collect the lymphocyte. The cell pellet was further suspended in 100 μl Ca^2+^ and Mg^2+^ free PBS and further processed for comet assay. Single cell alkaline gel electrophoresis was performed as described earlier [21], [22]. Analysis of the slides was done same day and cells were scored using image analysis system (Komet 5.5; Kinetic Imaging, Liverpool, UK) attached to an Olympus (CX41) fluorescent microscope (Olympus Optical Co, Tokyo, Japan) and a COHU 4910 integrated CC camera equipped with 510–560 nm excitation and 590 nm barrier filters (COHU, San Diego, CA, USA). Images from 50 cells (25 from each replicate slide) were analysed. Migration of DNA from the nucleus i.e. tail length was measured as the main parameter to assess lymphocyte DNA damage.

## Result and Discussion

### UV–Visible Spectroscopy

**Figure 1:**
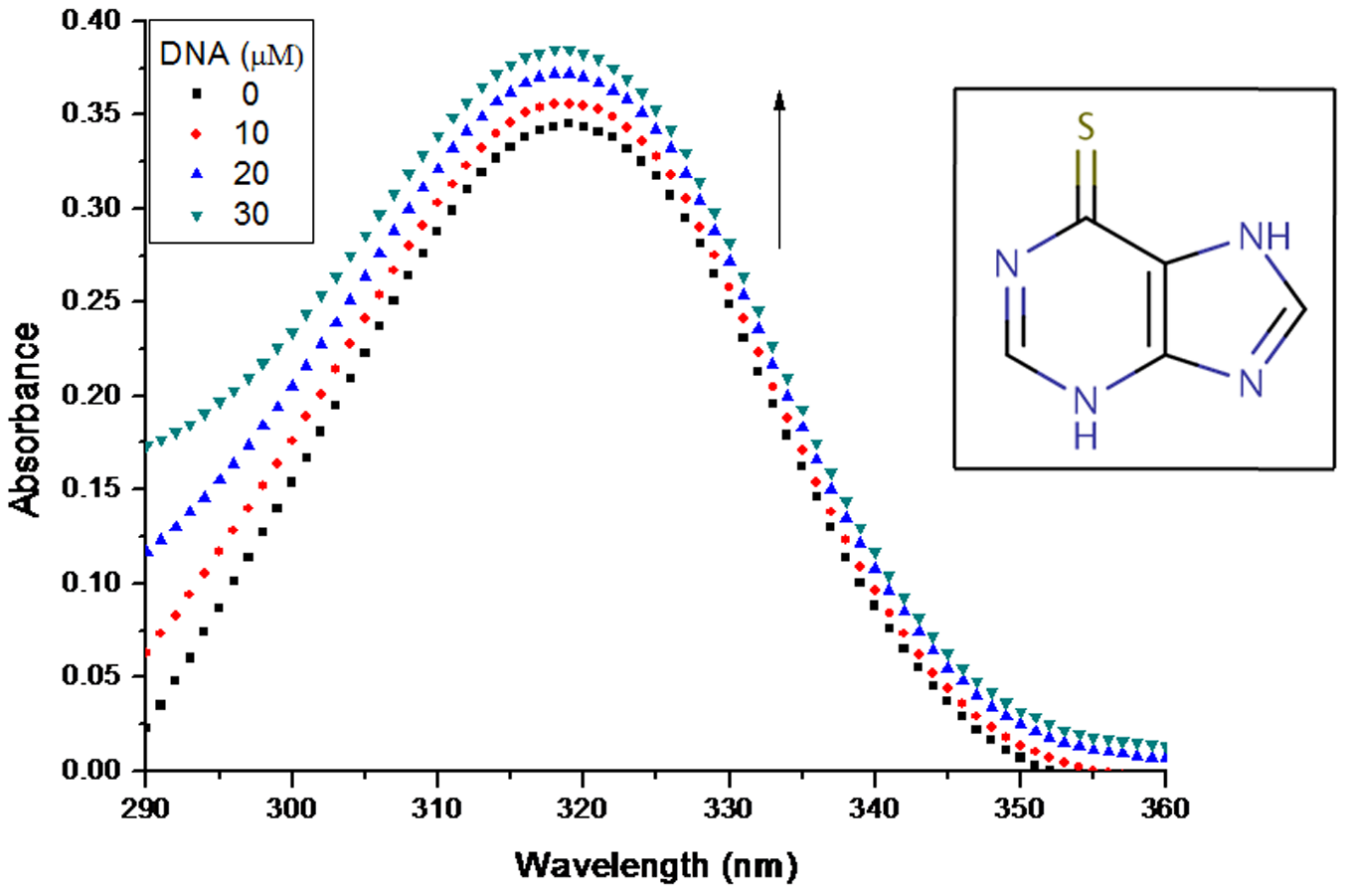
Interaction of 6MP with CT-DNA. UV-visible absorption spectra of 6MP (25 μM) in presence of increasing concentrations of CT-DNA in Tris-HCl buffer (pH 7.2). Hyperchromism was observed with increasing concentration of CT-DNA confirming the interaction of 6MP and DNA. Structure of 6 Mercaptopurine is shown in the inset.

Interaction of small molecules with DNA is easily studied and interpreted using UV-visible spectroscopy. We exploited this technique to investigate 6MP-DNA interaction. 6MP alone shows maximum absorption near 320 nm. On subsequent addition of CT-DNA, hyperchromism was observed with no apparent shift in the position of maximum absorption peak (Figure 1). It is well known that intercalation of small molecules into the DNA helix results in bathochromic shift as well as hypochromism [23]. Since bathochromic shift or hypochromism was not observed on 6MP and CT-DNA interaction, intercalation mode of binding can be excluded in case of 6MP. However, the absence of any clear isobestic point in the 6MP-DNA spectra indicates that more than one type of binding may be present or 1∶1/drug: DNA stoichiometry is not maintained during the process [24]. The exact mode of interaction cannot be established merely by this technique, hence further experiments were required to explore the binding mode.

### Steady State Fluorescence

**Figure 2:**
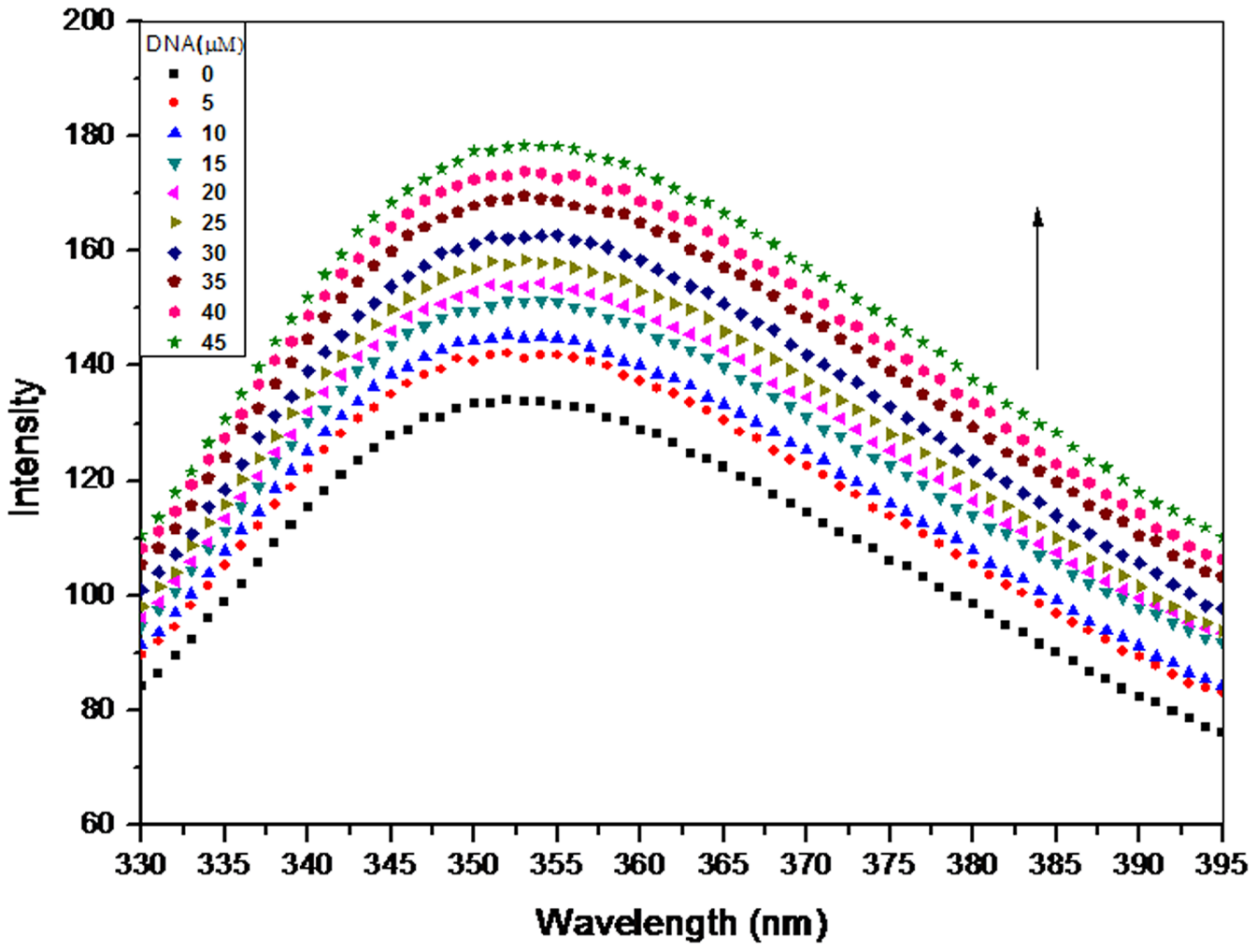
Interaction of 6MP with CT-DNA studied using fluorescence spectroscopy. Fluorescence emission spectra of 6MP (50 μM) in the presence of increasing concentrations of CT-DNA. Increase in the fluorescent intensity was observed with increasing DNA concentration. Excitation wavelength was 280 nm and emission was recorded as shown in figure.

**Figure 3:**
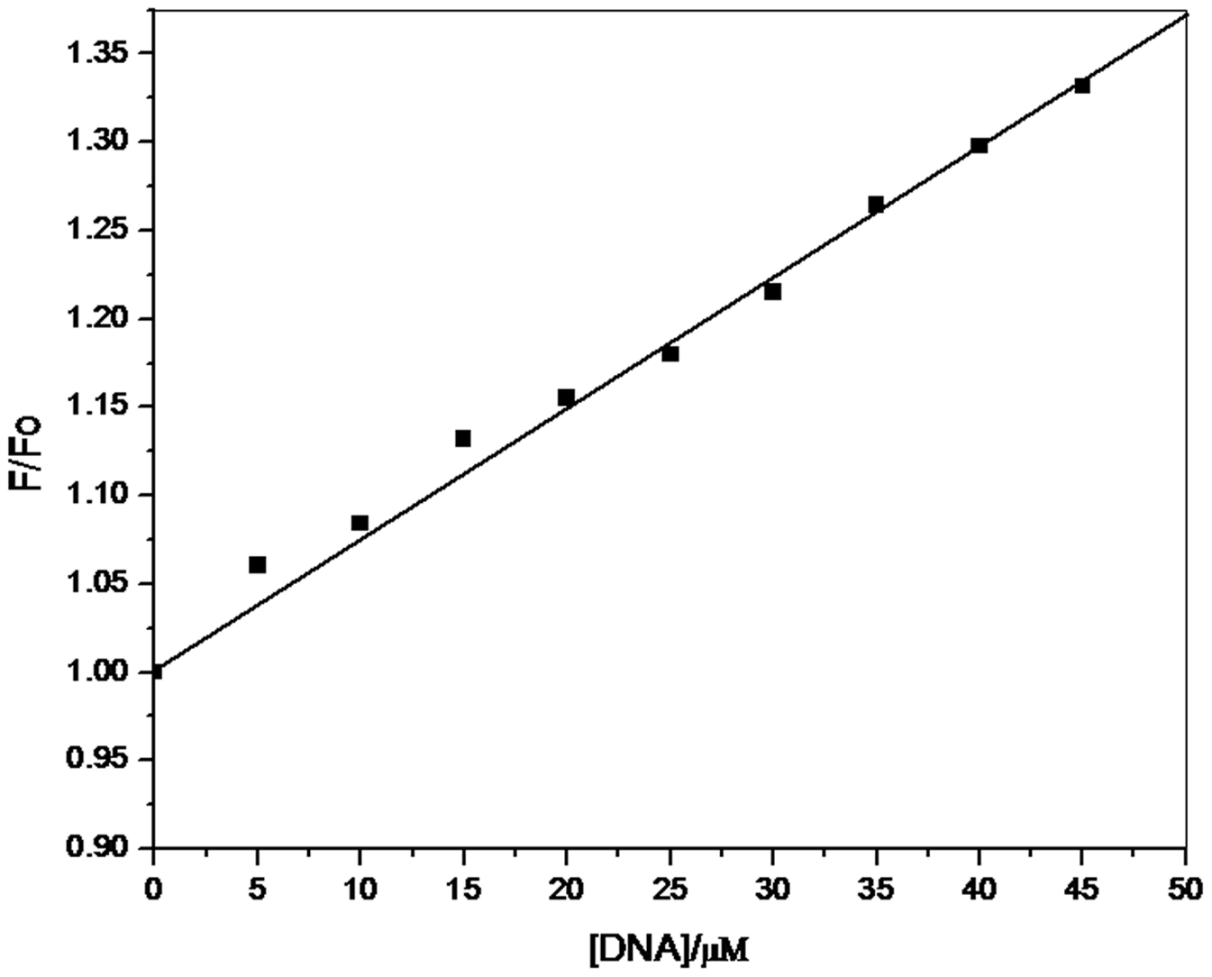
Stern-Volmer plot for interaction of 6MP with CT-DNA. The fluorescent intensity was found to be directly proportional to DNA concentration. Binding constant of 7.8×10^3^M^−1^ was obtained from the slope.

To further elucidate the interaction of 6MP with CT-DNA, steady state fluorescence was employed in our study. Since the endogenous fluorescence property of DNA is poor, we studied the fluorescence spectra of 6MP in all subsequent studies. Emission spectra of 6MP in 10mM Tris-HCl, pH 7.2, showed a broad unstructured peak with maxima around 350 nm (Figure 2). On addition of CT-DNA, enhancement in the fluorescence yield occurred with no detectable shift in the absorption peak position. This hyperchromism establishes the binding interaction of 6MP with CT-DNA. To further understand the interaction, ratio of peak fluorescence intensity in presence and in absence of CT-DNA (F/F_0_) was plotted as a function of DNA concentration (Figure 3). The plot indicated that the fluorescent intensity is directly proportional to the CT-DNA concentration. Further, Ksv (Stern–Volmer quenching constant) was calculated since it is considered as a measure for efficiency of fluorescence quenching by DNA. Ksv was obtained from the slope of Figure 3 and was calculated to be 7.48×10^3^ M^−1^, which was much lower than the other classical intercalators [25]–[26] and hence indicating less possibility of intercalation of 6MP with CT-DNA. Thus, 6MP is suggested to interact with DNA via non-intercalative binding mode.

### Competitive Displacement Assay

**Figure 4:**
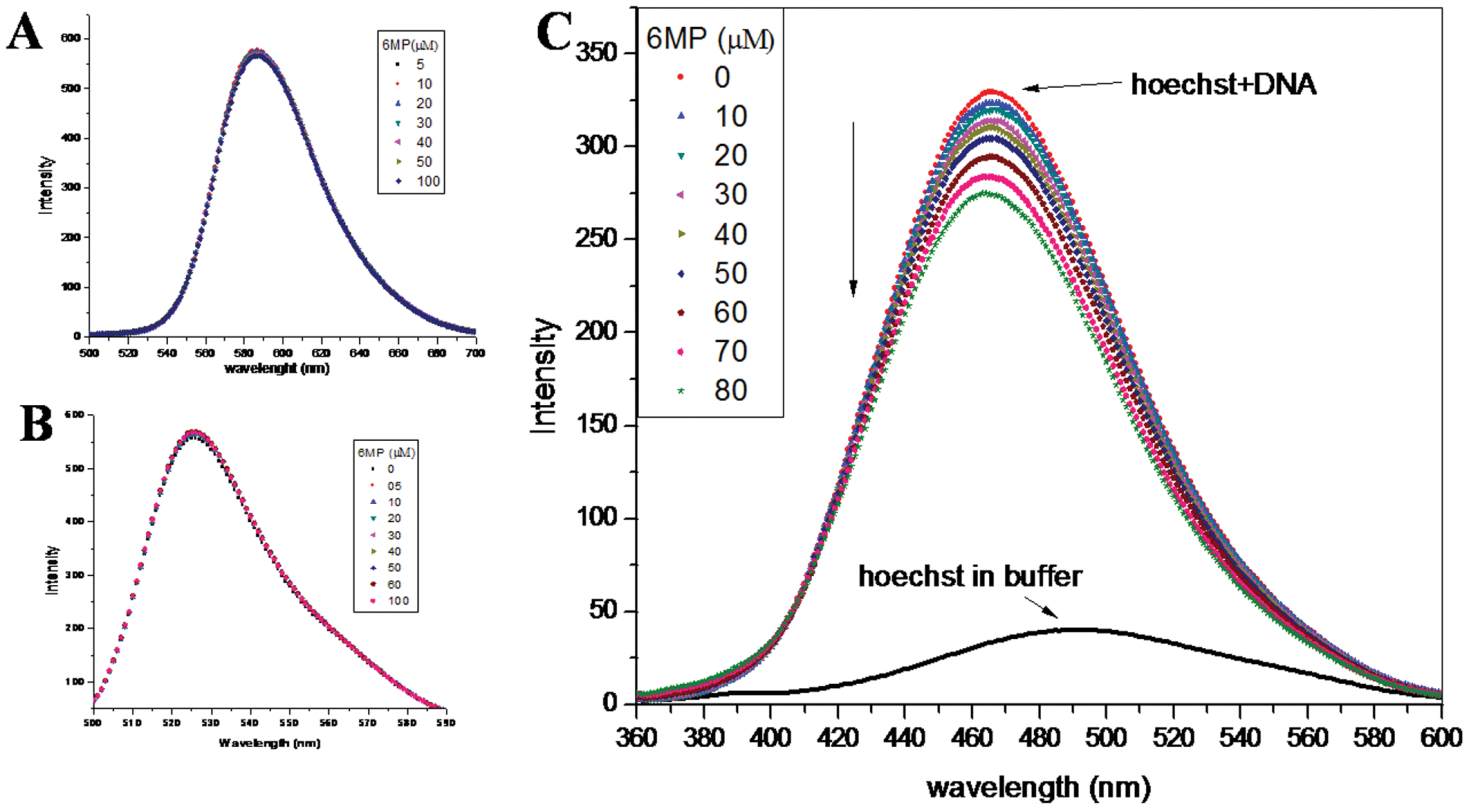
Competitive displacement assays. (**A**) Fluorescence titration of CT-DNA and EB (intercalator) complex with 6MP. EB-DNA complex was excited at 471 nm and emission spectra were recorded from 500–700 nm. No change in fluorescence intensity was recorded with addition of increasing concentration of 6MP (**B**) Fluorescence titration of CT-DNA and AO (intercalator) complex with 6MP. CT-DNA-AO complex was excited at 471 nm and emission spectra were recorded from 500–600 nm. No change in fluorescence intensity was recorded with addition of increasing concentration of 6MP. (**C**) Fluorescence titration of CT- DNA and Hoechst (groove binder) complex with 6MP. Fluorescence intensity decreases with subsequent addition of 6MP. CT-DNA-Hoechst complex was excited at 343 nm and emission spectra were recorded from 360–600 nm.

DNA binding dyes are extensively used to study the mode of drug-DNA interaction. Binding of such dyes to DNA are well studied and their binding mode is well established. Any small molecule that competitively replaces a bound dye from DNA helix is expected to bind the DNA in similar fashion as the bound dye [27]–[30]. Thus any change in fluorescence intensity of dye-DNA complex on addition of small molecule is easily interpreted. EB is a well known probe that binds to the DNA in intercalative fashion [27]. Since EB works as an excellent spectral probe to investigate the binding mode of drug with DNA, it was used to confirm the mode of binding of 6MP to CT-DNA. With continuous addition of 6MP to the system, there was no significant change in the fluorescence intensity (Figure 4A). This suggested that 6MP does not replace EB from CT-DNA helix as 6MP binds to CT-DNA in non-intercalative mode. To further confirm the binding mode, we used AO in a similar competitive replacement assay. AO is a classical intercalating dye [28] and it was not replaced by 6MP as expected (Figure 4B). In another experiment, Hoechst 33258, which binds to the minor groove of double stranded B-DNA [29], was used to study competitive replacement by groove binders. Hoechst 33258 on binding with DNA showed enhancement in the fluorescence intensity [30]. Groove binding molecules are able to displace Hoechst 33258 from the minor groove of DNA helix, resulting in decreased fluorescence intensity of DNA-Hoechst system. On addition of 6MP, the fluorescent intensity of Hoechst-DNA system was found to decrease as 6MP could replace the groove bounded dye from the CT-DNA (Figure 4C). This further suggested the binding mode of 6MP to be groove binding rather than intercalation.

### Iodide Quenching Studies

**Figure 5:**
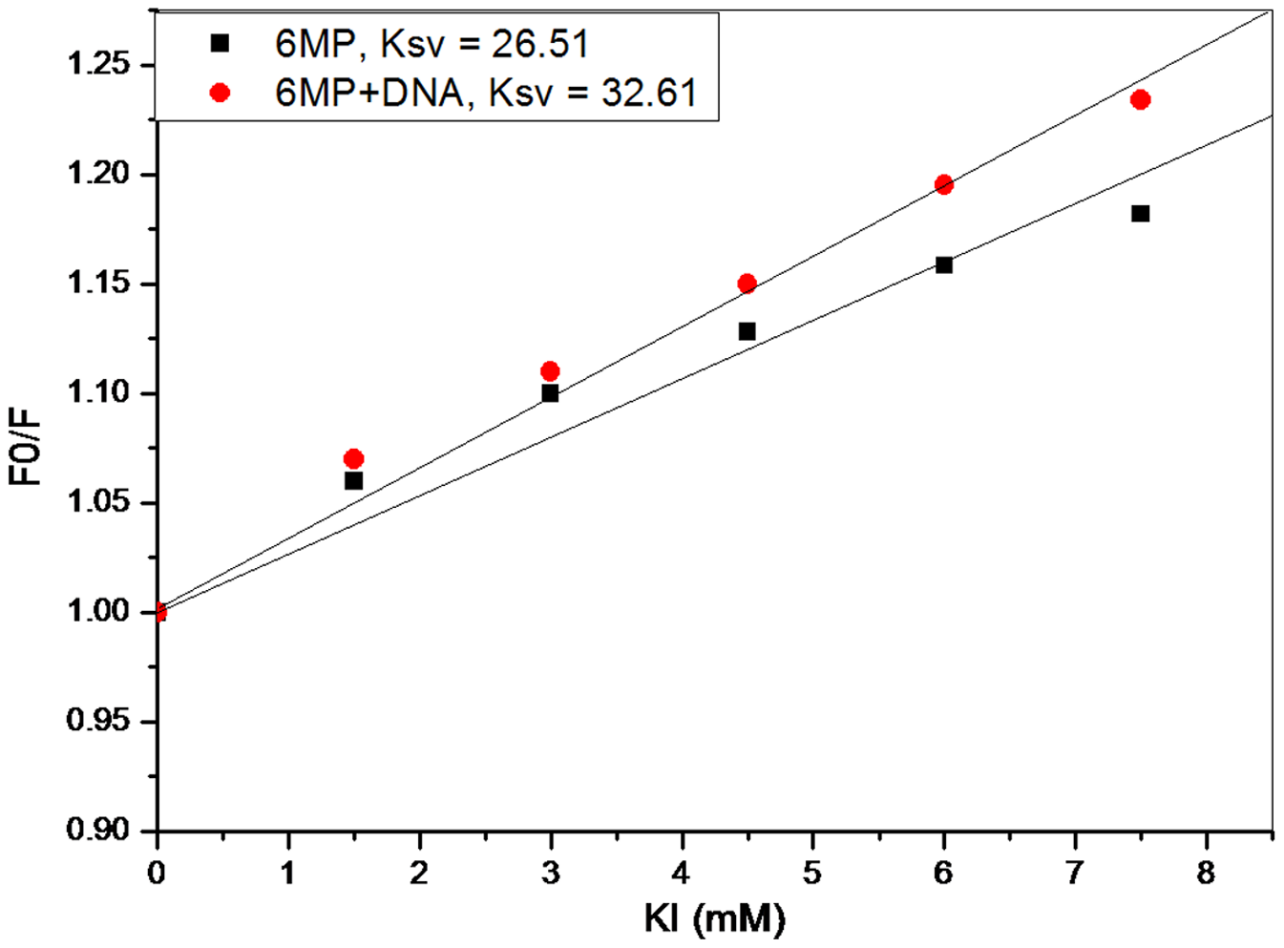
KI quenching experiment. Stern-Volmer plot for fluorescence quenching of 6MP (50 μM) by KI in absence and presence of CT-DNA (100 μM). Quenching of 6MP fluorescent intensity was done using KI in absence and presence of CT-DNA and quenching constant was calculated in both the case. Difference in Ksv value was further used to investigate the binding mode of 6MP and DNA.

Iodide ion quenching experiments provide great help in deducing the binding interaction of drug with DNA [31]–[33]. Iodide ions, being negatively charged, can effectively quench the fluorescence of small molecules in an aqueous medium. However, in presence of DNA, iodide ions are repelled by negatively charged phosphates present in DNA backbone. Any small molecule intercalated into the DNA helix is well protected as the approach of anionic quenchers towards such molecule is restricted. However, this is not the case with electrostatic binding and groove binding molecules which are exposed to the external environment and are easily approachable for quenchers even in presence of DNA [31]. The relative accessibility of small molecules to anionic quencher in free medium and in presence of DNA is studied by calculating Ksv using Stern-Volmer equation
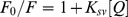
where F_0_ and F are the highest fluorescence intensity in the absence and presence of the anionic quencher [Q]. K_sv_ is Stern-Volmer quenching constant calculated from the slope of [F_0_/F] vs [Q] plot. K_sv_ obtained in absence and presence of DNA environment signifies the binding mode of drug. Relative decrease in K_sv_ in presence of DNA occurs in case of intercalation, however it remains unchanged when interaction is electrostatic or groove binding. As seen in Figure 5, KI could effectively quench the fluorescence of 6MP in a buffer solution and a K_sv_ value of 26.51 M^−1^ was obtained. However, in presence of CT-DNA, there was an increase in the K_sv_ value to 32.61 M^−1^. Since, earlier experiments suggested for a groove binding mode of interaction between 6MP and DNA, relatively similar K_sv_ value was expected in KI quenching studies. However, this unexpected increase in Ksv value, decrease in fluorescence yield, can be explained by involving the role of ionic strength. Firstly, on addition of KI there is increase in the ionic strength in the medium resulting in the release of DNA bound 6MP. Since the fluorescence intensity of free 6MP is less than 6MP-DNA complex, there is a decrease in fluorescence yield. Also, KI effectively quenches the fluorescence of free 6MP in solution. Hence two factors operate together resulting in enhanced quenching of fluorescence intensity by KI in presence of DNA causing an increased K_sv_ value. Thus, it can be confirmed that groove binding mode of interaction occurs between 6MP and DNA.

### Effect of Ionic Strength

**Figure 6:**
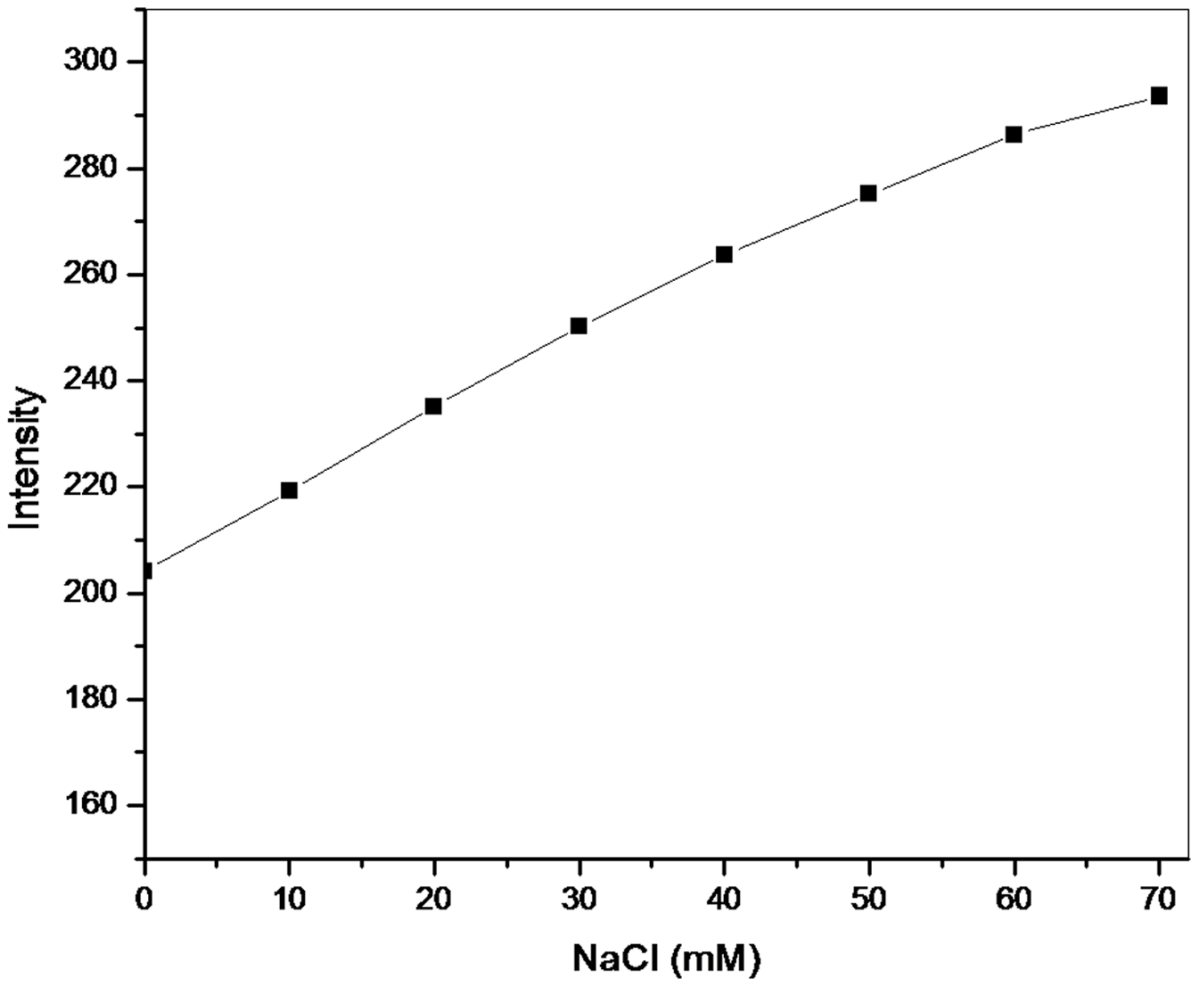
Role of ionic strength. To study the role of electrostatic effect on 6MP-DNA binding, NaCl was used. Maximum emission intensity plot of 6MP-DNA was plotted with increasing concentration of NaCl (0–70mM). Excitation wavelength was 280 nm. Increase in fluorescence intensity suggests for a possible electrostatic interaction between 6MP and CT-DNA.

Studying the effect of ionic strength is also a resourceful method to differentiate the binding mode between small molecules and DNA. Generally, strong electrolyte such as NaCl is used where the addition of NaCl does not affect the fluorescence yield of drug alone. In presence of DNA, Na^+^ partly neutralizes the negative charges of DNA phosphate backbone resulting in reduced electrostatic repulsion between them. The electrostatic attraction between small molecule and DNA surface is weakened by the addition of Na^+^. In case of surface binding molecules, the electrostatic binding takes place out of the groove, addition of NaCl will weaken the interaction resulting in the weakening of quenched fluorescent intensity [34]. In our study, addition of NaCl to 6MP-DNA complex (Figure 6) increased the fluorescence intensity. This observation could be explained on the basis that the negative charge of DNA phosphate backbone is neutralised with the addition of NaCl causing negatively charged 6MP to further interact with DNA resulting in enhanced fluorescence intensity. This enhancement may have masked the probable decrease in the electrostatic interaction on increasing the ionic strength. Thus, electrostatic interaction between 6MP and DNA cannot be ruled out.

### Circular Dichroism Studies

**Figure 7:**
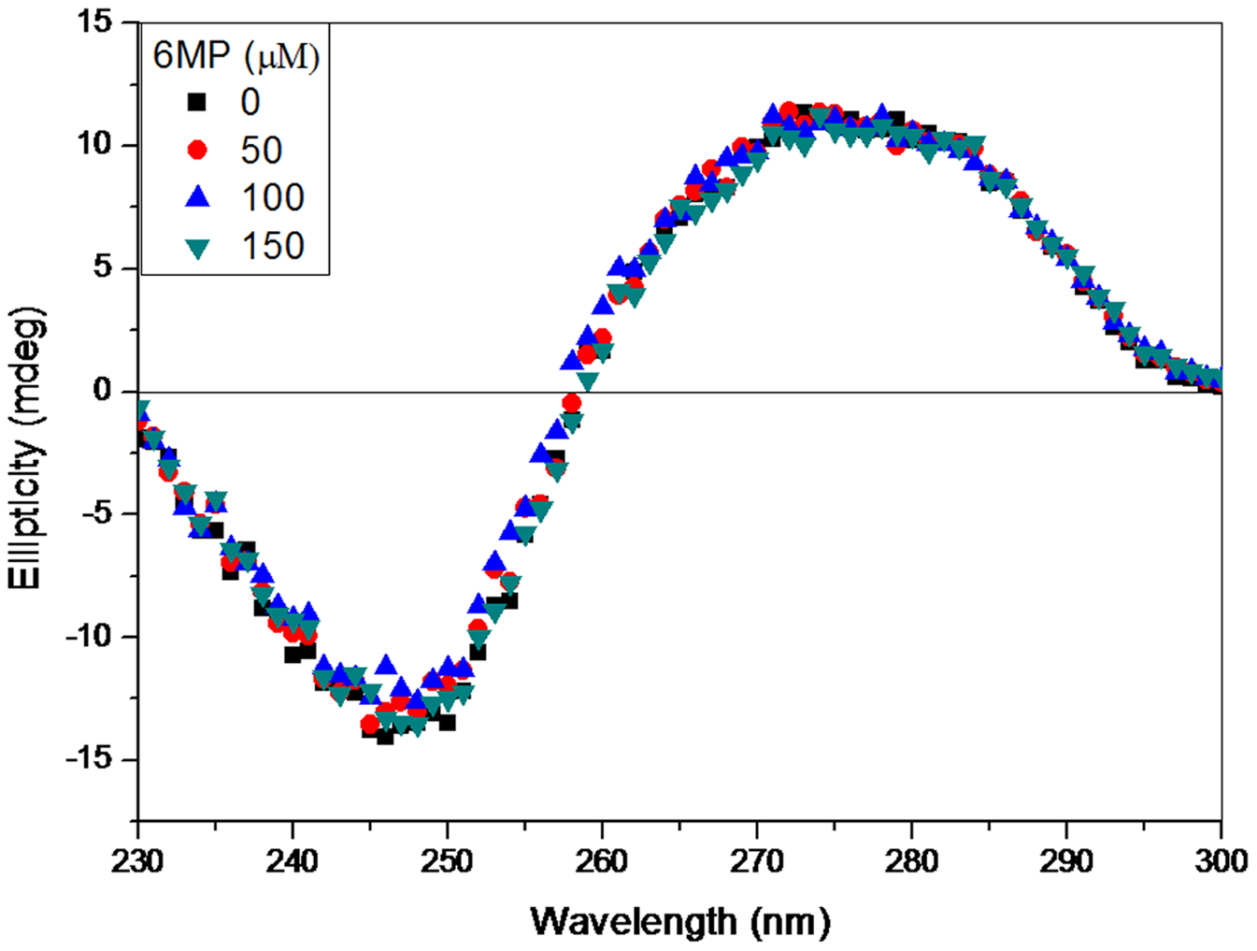
Effect of 6MP on CD spectra of CT-DNA. CD spectra of CT-DNA (30 μM) in 10mM Tris-HCl (pH 7.2) with the addition of varying concentration of 6MP. Each spectrum was obtained at 25°C with a 10 mm path length cell.

CD spectroscopy is a sensitive technique to detect any alteration in the DNA backbone. Non covalent DNA-drug interactions affect the structure of the DNA, and hence alter their intrinsic CD spectral behaviour [35]. As seen in figure 7, the CD spectrum of free CT-DNA in Tris-HCl at pH 7.2 showed a positive peak at 277 nm and a negative peak at 245nm which represents the right-handed B-form with 10.4 base pairs per turn. In CD spectrum of CT-DNA alone, there are four major bands i.e. at 277 nm (positive), 243 nm (negative), 223 nm (positive) and 213 nm (negative). The positive band at ∼277 nm is due to base stacking and helicity is responsible for a negative band at ∼243 nm which is a characteristic of right-handed B form DNA [36]–[38]. These bands are considered to be highly sensitive toward interaction of small molecules with DNA [39], [40]. It is known that binding of a molecule with DNA may stabilize or destabilize the DNA structure. Secondary structure of DNA is altered by intercalation with small molecules [41], [42]. However, minor groove binders do not perturb the CD spectrum of DNA significantly. In order to obtain further information about the binding of 6MP to DNA, we recorded the CD spectra of CT-DNA with increasing concentration (0–150 μM) of 6MP (Figure 7). There was no noticeable change in the CD spectra confirming the absence of intercalation of 6MP within DNA strands.

### Viscosity Measurement

**Figure 8:**
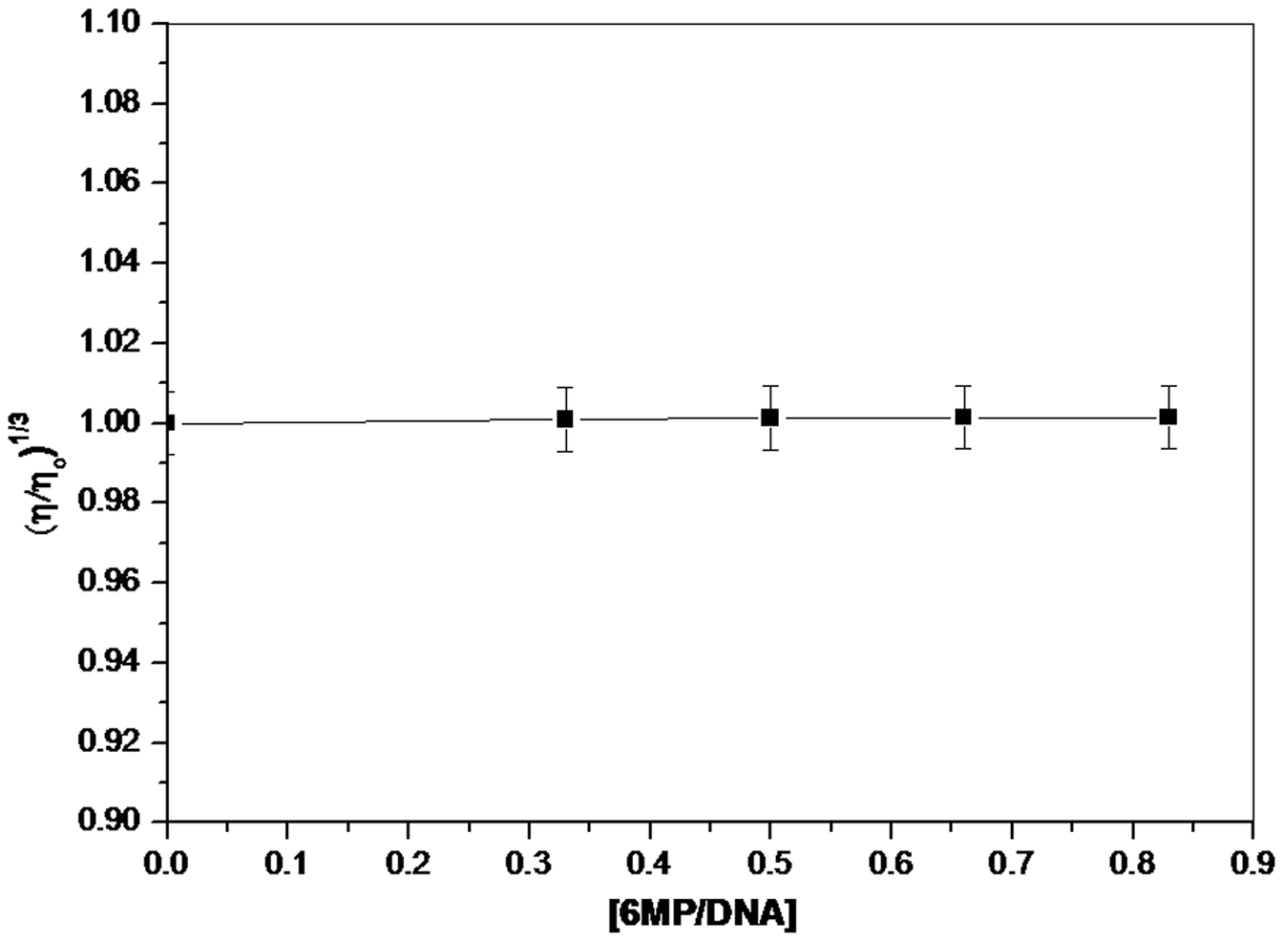
Effect of increasing the concentration of 6MP on the viscosity of CT-DNA. The concentration of CT-DNA was kept constant (100 μM) with increasing amount of 6MP. Values reported are mean of three independent experiments.

Viscosity measurement of drug-DNA complex provides reliable evidence to study the mode of interaction. In case of intercalative mode of binding, length of DNA helix is increased due to separation of base pairs resulting in increased viscosity of DNA [43], [44]. On the other hand, if the drug interacts with DNA via electrostatic/groove binding mode, viscosity of DNA solution does not change significantly [45]–[47]. A plot of (η/η_0_)^1/3^ versus [6MP]/[DNA] was obtained to study any change in viscosity of CT-DNA solution in presence of 6MP. As seen in Figure 8, with continuing addition of 6MP to CT-DNA solution, viscosity of CT-DNA solution remains the same. This confirms that 6MP binds to DNA via external binding mode and hence does not intercalate into DNA helix.

### Molecular Docking

**Figure 9:**
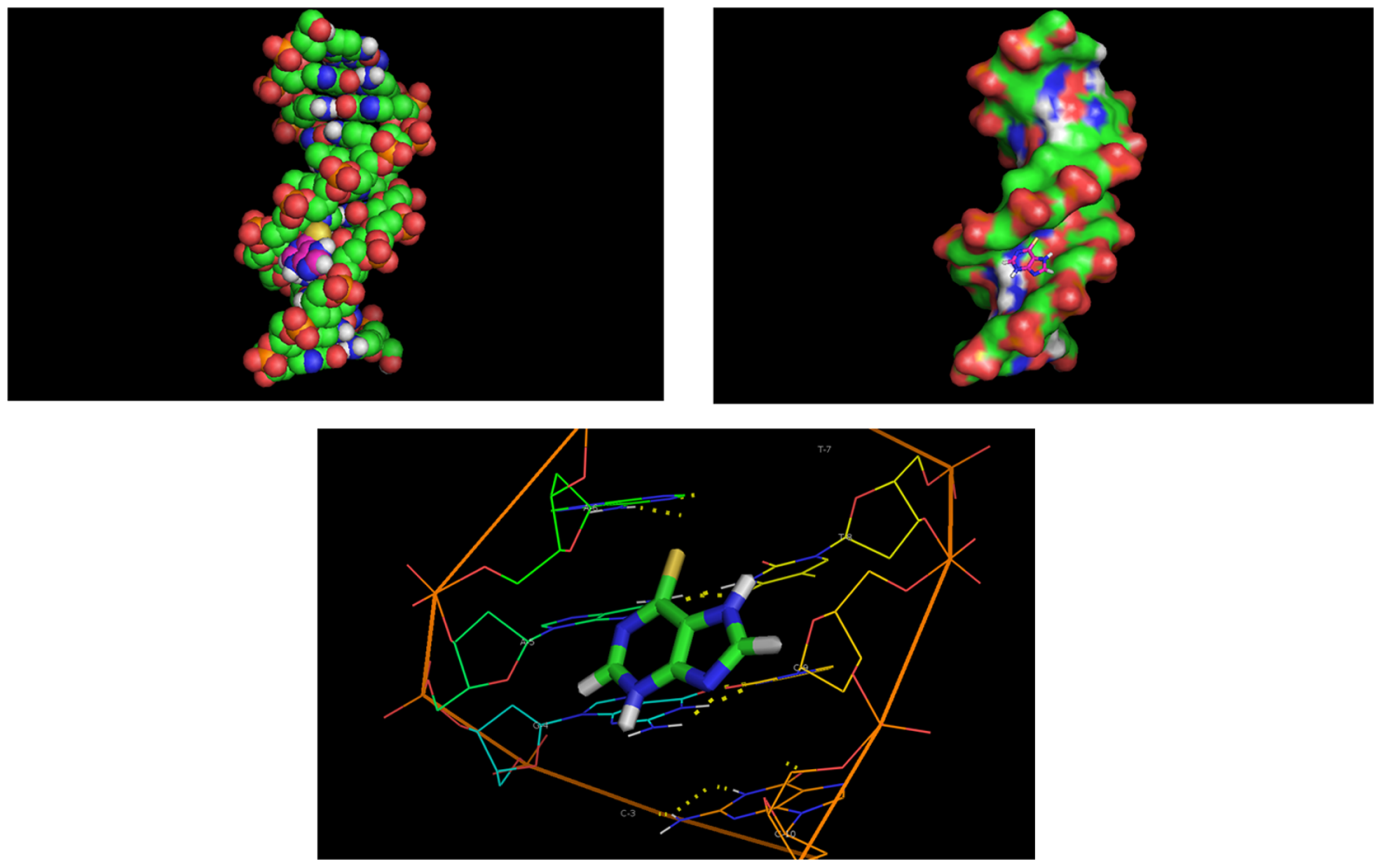
Molecular docked structure of 6MP complex with DNA. Dodecamer duplex sequence (CGCGAATTCGCG)_2_ (PDB ID: 1BNA) was used in the docking studies. The binding energy of the complex system was found to be −116.97 kJ/mole.

Molecular docking techniques are an attractive scaffold to understand the drug–DNA interactions in rational drug design, as well as in the mechanistic study by placing a small molecule into the binding site of the target specific region of the DNA mainly in a non-covalent fashion. Structure of drug is made flexible to attain different conformations in order to predict the best fit orientation, and the best energy docked structure is analyzed. Figure 9 shows the minimized orientation of minor groove interaction of 6MP with DNA. The minimized conformation of 6MP sitting in the groove of the sequence d(CGCGAATTCGCG)_2_ does not show hydrogen bonding with the base pairs of dodecamer. The binding energy of the complex system was −116.97 kJ/mole. Even in the presence of similar charge on 6MP and DNA backbone, the negative value of binding energy indicated binding potential of 6MP with DNA. The docking result further supported the groove binding mode between 6MP and DNA that was earlier obtained with the help of spectral techniques.

#### Photo-induced ROS generation and DNA damage by 6MP

**Figure 10:**
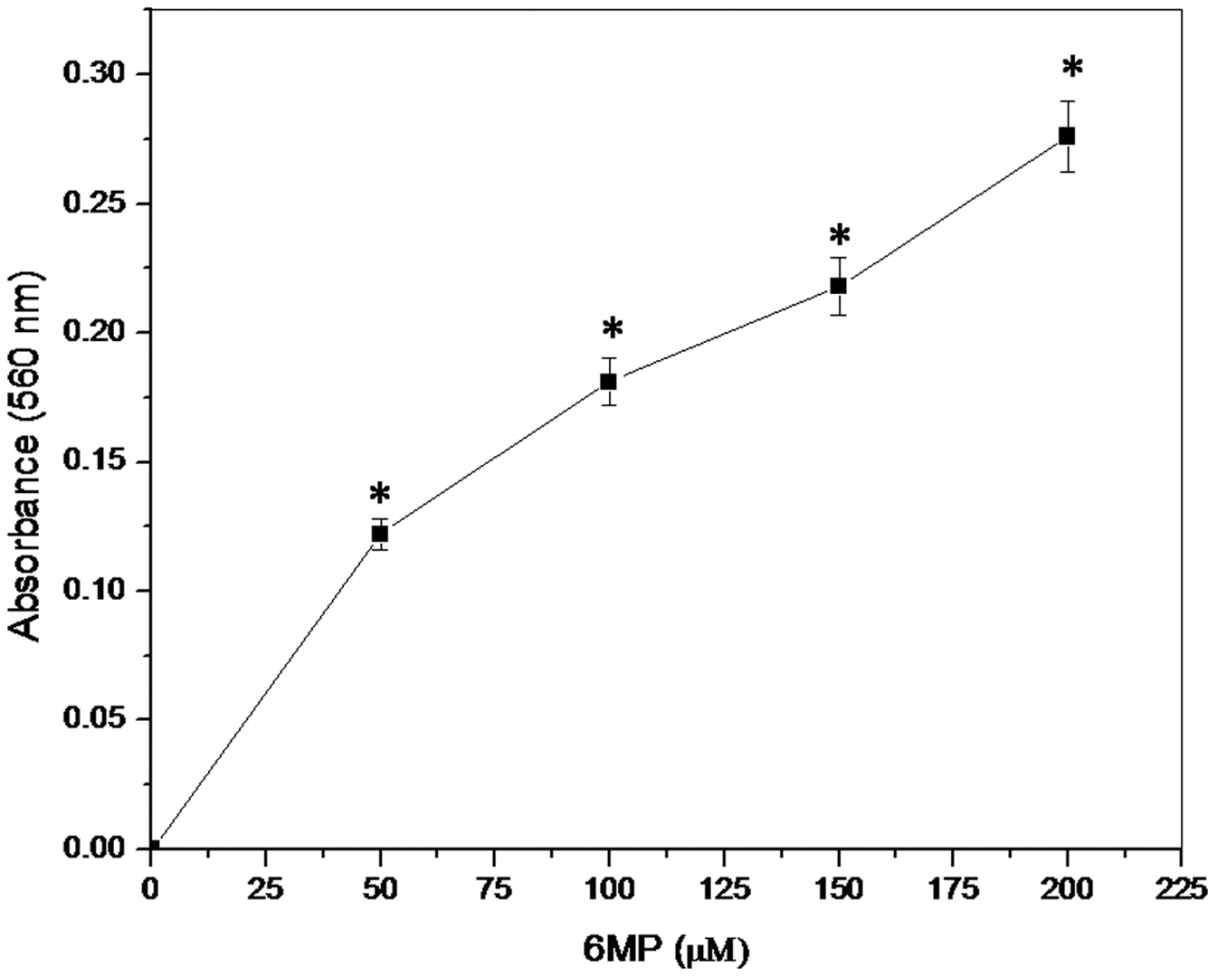
6MP induced generation of superoxide anion. Concentration dependent photo generation of superoxide anion by 6MP. Indicated concentration of 6MP was exposed to white light for 1h at RT and absorbance was measured at 560±SEM of three independent experiments. *p value <0.01 when compared to control.

6MP induced DNA damage in presence of light was studied using various techniques. In NBT assay, upon induction with white light 6MP produces superoxide anion, these superoxide anion reduce NBT via a one-electron transfer reaction, producing partially reduced (2e^−^) monoformazan (NBT^+^) as a stable intermediate, whose formation can be recorded spectrophotometrically at 560 nm. With increasing concentration of 6MP, there was a consistent increase in the generation of superoxide anions in presence of white light (Figure 10).

**Figure 11:**
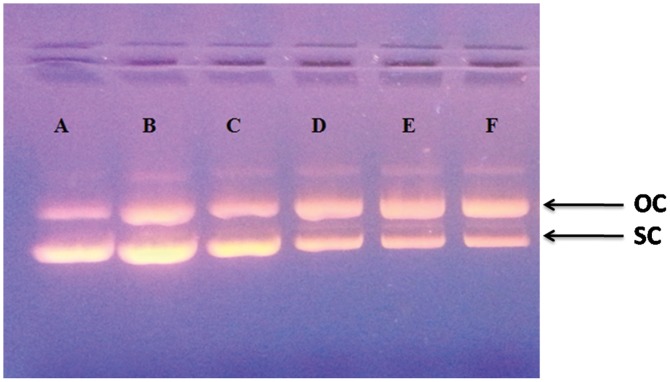
6MP induced damage to plasmid DNA in presence of light. Agarose gel electrophoresis pattern of ethidium bromide stained pBR322 DNA after the treatment with 6MP in presence of white light. Lane ‘**A**’ depicts the ‘Control’ which contain only plasmid DNA. The concentrations of 6MP in lanes ‘**B–F**’ was 100, 200, 300, 400 and 500 μM respectively. Arrows indicating OC and SC on the right represent the open circular and supercoiled forms of plasmid DNA.

**Figure 12:**
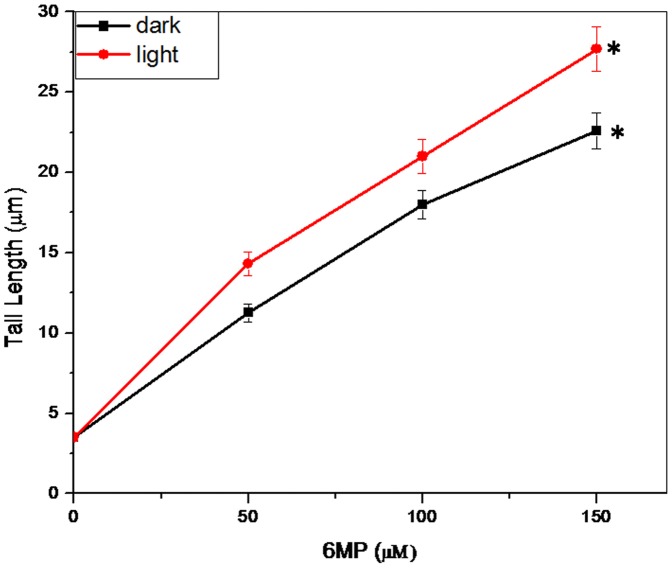
6MP induced human lymphocyte DNA breakage. Comet tail length obtained after treatment with 6MP in dark and light. Values reported are ±SEM of three independent experiments. *p value <0.01 when compared to control.

To further study DNA damaging ability of 6MP induced ROS in presence of light, plasmid nicking assay was performed. Plasmid nicking assay is a sensitive method to detect DNA damage directly caused by any interacting small molecule/drug. Various concentrations of 6MP used in the present study in absence of light did not cause any nick in double stranded plasmid DNA. However, in the presence of white light, there was an increase in formation of open circular form of plasmid DNA due to nicking caused by 6MP induced ROS. As seen in Figure 11, increase in the intensity of the open circular form and decrease in the intensity of a DNA band corresponding to super coiled DNA depicts the plasmid DNA strand breaks activity. It is well documented that ROS such as singlet oxygen and superoxide act as major toxic mediators in the upstream of drug-induced phototoxic cascades [48]. To further study the 6MP induced DNA damage in the cell, single cell gel electrophoresis (comet assay) was performed using human lymphocyte. Comet assay is a simple, rapid and sensitive method for detecting DNA damage [21]. It is also very often used to detect DNA cross links, presence of alkali labile sites and incomplete excision repair sites apart from finding great application in genotoxicological studies [22]. Cells treated with test compounds are subjected to unwinding and electrophoresis at neutral pH (to detect double strand breaks) or alkaline pH (to detect single strand breaks) after cell lysis in high salt concentration. In presence of electric field, DNA migrates towards the positive electrode and a comet like pattern is observed after final processing. Comet consists of a head and a tail that includes undamaged and damaged DNA respectively. In absence of light, 6MP caused little damage to lymphocyte DNA. This observation can be attributed to the presence of various metals ions associated with DNA. In a study (result not shown) we have shown the redox cycling of copper, one of the metal ions associated with DNA, by 6MP and subsequent generation of ROS that damage DNA. However, in presence of light, degree of damage was high as increase in tail length was observed (Figure 12). This is possible due to enhanced ROS generation by 6MP in presence of light.

In conclusion, we have studied the mode of interactions between 6MP and DNA using various approaches and confirmed that there could be mixed type of binding interactions. Groove binding of 6MP to DNA was confirmed while role of electrostatic interactions cannot be ruled out. However, 6MP does not intercalate into the strands of DNA. In absence of light, under observed concentration, 6MP did not cause nick in plasmid DNA. However, DNA damage was observed when 6MP-DNA was exposed to light in plasmid nicking assay. In case of comet assay, there was a noticeable damage to DNA even in absence of light. However, the damage was enhanced to a greater extent in presence of light. Our present study clearly demonstrated the photogenotoxic action of 6MP probably mediated by ROS. This study could also be useful to classify drug as phototoxic or non-phototoxic and also provide a deep insight in determining the mechanism of action of various drugs.
